# Transgenerational improvements following child anxiety treatment: An exploratory examination

**DOI:** 10.1371/journal.pone.0212667

**Published:** 2019-02-28

**Authors:** Kristen Lavallee, Kathrin Schuck, Judith Blatter-Meunier, Silvia Schneider

**Affiliations:** 1 Ruhr-University Bochum, Bochum, Germany; 2 Praxis Beim Spalentor, Basel, Switzerland; Temple University, UNITED STATES

## Abstract

**Background:**

The present study conducted secondary analyses of a randomized controlled trial to examine the transgenerational relationship between cognitive-behavioral therapy for child Separation Anxiety Disorder (SepAD) and the mental health of parents. Symptoms of anxiety and depression were compared before and after child treatment between parents of children treated for SepAD and parents of healthy children, who did not receive any treatment.

**Methods:**

One hundred and seven children aged 4–14 years with SepAD received one of two cognitive behavioral treatment programs for SepAD (TAFF; *TrennungsAngstprogramm Für Familien*; English: Separation Anxiety Family Therapy or CC; Coping Cat). Their parents (N = 189; 101 mothers and 88 fathers) were assessed at baseline and post-treatment for symptoms of separation anxiety, general anxiety, and depression. A comparison group of parents (N = 74; 42 mothers and 32 fathers) of 45 children without SepAD, who did not receive any treatment, were also assessed.

**Results:**

Results indicated a significant interaction effect between group and time on mothers’ depression and separation anxiety, indicating that maternal symptoms of depression and separation anxiety improved in the child treatment condition in comparison to mothers of healthy children. There was no significant improvement in parental pathology levels among fathers of children treated for SepAD.

**Conclusions:**

Treatment for child SepAD may have subsequent positive effects on mothers’ own levels of separation anxiety and depression, though the mechanisms are yet unknown. Future studies are needed that test the transgenerational effect of child SepAD treatment on parental mental health as the primary research question.

## Introduction

Separation Anxiety Disorder (SepAD) is one of the most common anxiety disorders of childhood [1, 2]. Unique to SepAD among anxiety disorders [3], separation anxiety directly involves and centers on primary caregivers, as children with SepAD are specifically anxious about being apart from them. Etiological research indicates that parent behaviors and cognitions are associated with child´s SepAD, in that parents of anxious children have more dysfunctional beliefs and lower parenting self-efficacy than parents of non- or less anxious children [4], and that parents’ own pathology is related to child SepAD [5–7].

### Efficacy of treatment for child separation anxiety

Cognitive-behavioral therapy (CBT) is considered a well-established treatment for anxiety disorders in general, with an average remission rate of 56–69% [8–10], and mean pre-post treatment effect sizes of 0.58 (intent-to-treat) to 0.86 (completers only) in meta-analyses [9, 10]. One of the most widely-used catch-all anxiety treatment programs, *Coping Cat* (CC,[11, 12] and adaptations [13], have demonstrated good efficacy in treating a variety of childhood anxiety disorders, with between-group effect sizes of 0.87 across disorders (including SepAD) on general anxiety measures as compared to waitlist [11]. Recovery for children undergoing *Coping Cat* treatment ranges, as indicated by the percentage of children no longer meeting diagnostic criteria, between 59% [13] and 64–71% [11, 12], with gains well-maintained over time [14].

Despite the well-established efficacy of general CBT treatment programs for child anxiety disorders, a substantial number of children do not sufficiently benefit from current treatment approaches [9]. Approximately 30–40% of treated children will experience a return of anxiety (i.e., relapse) after successful treatment [15]. Therefore, some treatment programs have attempted to improve remission and relapse rates by adding disorder-specific as well as family-focused treatment components to general CBT programs. A disorder-specific, family-focused treatment program for child SepAD is the TAFF-program (German: *TrennungsAngstprogramm Für Familien*; English: Separation Anxiety Family Therapy) [16, 17]. TAFF incorporates disorder-specific materials that are appropriate for children aged between 5 and 13 years and includes a parent training (targeting parenting behavior and parent-child interaction). The rationale for including parents is based on the embedded nature of separation anxiety within the context of the family. TAFF was found to be highly effective in comparison to a waitlist condition in 5–7 year-old children [16], with a remittance rate of 76.2%, and large effect sizes (d = 0.98 to 1.41). Indeed, effects obtained with the disorder-specific treatment for SepAD were larger than those reported in the meta-analysis of childhood treatment for anxiety disorders [9]. A second trial also demonstrated efficacy of TAFF in comparison to CC (*Coping Cat;* [11, 12]) among 8–13 year-old children, with no significant differences between these treatment programs [17].

### Effects of child treatment on parents’ pathology

A more recent research question in the literature involves the transgenerational effects of therapy on the family members of patients in treatment. Environmental experiences can modify an individual’s social, emotional, and cognitive traits and behaviors during an individual’s lifetime [18], and thus it would seem that a change in a family member’s behavior or emotional state might have an effect on an individual’s own well-being. Indeed, the family system is one of the most important social environments and family influences are established predictors of mental health and mental health problems [19]. Further, recent research attending to the dyadic nature of personal growth, indicates that personal characteristics co-develop within relationships over time [20]. That is, change in one family member or friend can lead to changes in that individual’s spouse or friend [21, 22], with crossover between work and family as well [23].

With regard specifically to anxiety disorders, research has established substantial evidence for familiality and heritability. Children of parents with an anxiety disorder are more likely to develop anxiety disorders themselves compared to children of healthy parents [24]. Correspondingly, parents of children with an anxiety disorder show increased levels of anxiety themselves. Shared genetic factors and environmental experiences may explain cognitive, emotional, and behavioral similarities between family members. According to Social Learning Theory [25], reciprocal interactions shape cognitive learning (acquisition of knowledge, beliefs, norms, and values) as well as behavioral learning (social modeling or imitation) through continuous observations.

While a sizable body of research has demonstrated bidirectional adverse effects between parents and children with regard to emotional and behavioral problems [26–28], little research has addressed the potential beneficial effects of successful interventions on family members (i.e., transgenerational benefits of psychotherapy). Up to this point, the effects of one person in a family receiving therapy on the mental health of other family members are not well explored, except for a handful of studies. One study examined the transgenerational effects of parent psychotherapy for panic disorder on children, and found that parents’ treatment success was a significant predictor of better outcomes among their children with regard to anxiety sensitivity and agoraphobic cognitions six years after parents had completed treatment. Furthermore, it was shown that parental participation in treatment, regardless of the success of the treatment, had a significant positive effect on offspring [29]. This study indicates that parental psychotherapy for panic benefits not only the parent, but also their children, and suggests an intergenerational effect of anxiety disorder treatment.

As challenging child behavior is associated with parental distress and mental health problems [30], it may be expected that interventions improving child health may also have indirect effects on parental well-being. Surprisingly few studies have investigated potential beneficial effects of child treatment on parent mental health, but the few that have, do lend supporting evidence. For example, Reaven et al. (2015) [31] implemented a group therapy for managing anxiety in children with Autism Spectrum Disorders (ASD) and found that parental anxiety decreased among parents of children who responded to treatment. Similarly, Maughan and Weiss (2017) [32] examined the effects of CBT for children with ASD and found that parents whose children participated in CBT improved in symptoms of depression and emotion regulation abilities in comparison to parents of children on a waitlist for therapy. Kendall and colleagues (2008) [33], in a comparison of child-based and family-based therapy (as well as a third educational control condition) for children with anxiety disorders, also examined bottom-up effects of children’s treatment on parental wellbeing. In the sample of 161 mothers (61 with an anxiety disorder), they found post-psychotherapy of anxious children saw a reduction in maternal psychopathology, in that 43–45% of treated children’s mothers’ anxiety disorder (AD) diagnoses were no longer present at follow-up in the two therapy conditions. In this study, there was no significant difference between individual therapy and family-based therapy conditions. Mothers who participated in the educational control condition were reduced by 20%, though this was also not significantly different from the therapy conditions. There were also no significant changes in fathers’ anxiety disorders, perhaps due to fewer fathers being diagnosed with an anxiety disorder at the beginning of the study (24 fathers had an anxiety disorder pre-treatment). It could be that larger sample sizes and dimensional data (i.e., rating scales of anxiety) would be more sensitive to change than categorical anxiety diagnoses. In sum, there is some beginning evidence suggesting that parental mental health may change over the course of child treatment. Understanding these potential transgenerational effects of child treatment may inform our understanding of the variability in treatment outcomes among children (i.e., do children whose parents benefitted, also benefit more) and may eventually provide insight into how child treatment programs may best utilize parent involvement. With these implications and so little research to date, potential transgenerational psychotherapy outcomes are currently ripe for further exploration.

### The present study

The present study seeks to examine the transgenerational effects of treatment for child SepAD on the mental health of parents (i.e, symptoms of separation anxiety, general anxiety, and depression). To control for normal changes caused by time, analyses compared parents of children treated for SepAD (using one of two well-established treatment programs) to a quasi-control group of parents of healthy children, who did not receive any treatment. Previous studies have demonstrated the efficacy of CBT programs in reducing pathology among children, as well as post-child-therapy reductions in parent anxiety disorders. In the present study, it was hypothesized that child treatment would also improve mental health of parents, such as symptoms of anxiety and depression among parents, which often co-occur with child pathology. These effects were expected to be especially visible in mothers, as they may be more likely to have anxiety at pre-treatment.

## Method

### Study design

The present study uses combined data from two clinical trials (clinical trials registration identification number: NCT00255112; registry URL: http://www.clinicaltrials.gov) evaluating the efficacy of the TAFF treatment program for child SepAD. Detailed descriptions of these two trials are published elsewhere [16, 17]. In the first trial, children aged 5–7 years were randomly assigned to receive TAFF therapy or a waiting period, followed by TAFF therapy (Schneider et al., 2011). In the second trial, children aged 8–13 years were randomly assigned to either TAFF therapy or Coping Cat therapy [17]. All data of the treatment groups (both age groups and both TAFF and Coping Cat therapy) were combined for this study and only parental assessments were examined. Assessments took place at baseline, four weeks post-therapy, and one year post-therapy in all treatment groups. All available parents were invited to participate in therapy session when indicated in therapy protocols, and were invited to complete assessments.

Therapy was conducted by one fully qualified supervising psychotherapist and nine advanced clinical psychologists with specialized training in CBT. All therapists conducted treatments in both conditions. TAFF treatment was administered in 16 sessions consistent with the protocol [16]. CC treatment consisted of 16 sessions of child-focused therapy plus one wrap up session with the parents [11]. The present study was approved by the Basel Ethics Commission, the local ethics committee for medical research, and conducted at the University of Basel outpatient clinic from December 2004 to January 2009.

### Participants and procedure

Participants with SepAD were recruited through referrals from local service providers, and announcements in local newspapers, magazines, internet, schools, and family centers. Nonanxious participants were recruited via announcements in local newspapers, magazines, internet, schools, and family centers. Inclusion criteria for children with SepAD included meeting full diagnostic criteria for SepAD according to the Diagnostic and Statistical Manual for Mental Disorders–Text Revision (DSM-IV-TR) [34], age between 5 and 13 years old, German-speaking, not taking medication, and written parental informed consent and verbal child assent to randomized condition assignment and completion of psychological assessments. Families received free diagnostic assessment and treatment for their participation in the study. Inclusion criteria for children in the healthy control group included having no current DSM-IV-TR axis I diagnoses, having no lifetime SAD or SoP diagnoses, age between 5 and 13 years old, German-speaking, not taking medication, and written parental informed consent and verbal child assent to participation in psychological assessments.

In the first trial, forty-three children aged 5–7 years who met DSM-IV-TR criteria for SepAD were randomized to the immediate TAFF treatment condition (9 boys and 12 girls) or the delayed TAFF treatment condition, in which children received therapy after a waiting period equal to the duration of therapy (9 boys and 13 girls) [16]. In the second trial, sixty-four children aged 8–13 years who met DSM-IV-TR criteria for SepAD were randomly assigned to the TAFF treatment condition (15 boys, 16 girls) or the CC treatment condition (16 boys, 17 girls) [17]. Assignments to condition were randomized by a statistician using a computerized permuted block design [35], with assignments concealed until the time of participation. In the first trial, a total of four families dropped out and were lost to follow-up at 4-weeks post-treatment (three in the immediate treatment condition, one in the delayed treatment condition). In the second trial, all children underwent an initial 4-week waiting period before treatment. A total of 12 families dropped out and/or were lost to follow-up (7 in the TAFF condition and 5 in the CC condition). All available data were used in the analyses (with only baseline data for 13 families). The total combined sample in the present study consisted of parents of 107 children ages 5–13 who received treatment for SepAD (either TAFF or CC) and a control group of parents of 45 healthy children who did not receive any treatment. In total, 189 parents (101 mothers, 88 fathers) were in the child treatment condition (assessed at baseline and 4-weeks post-treatment) and 74 parents (42 mothers, 32 fathers) were in the control condition (assessed at baseline and the equivalent of four weeks post-treatment).

At baseline, child mean age was 9.1 years, *standard deviation (SD)* = 1.37. The 107 children in the SepAD therapy group had a mean age of 8.82, SD = 2.37, and the 45 children in the healthy control group had a mean age of 9.77 (SD = 2.27). Parents in the SepAD therapy group had a mean age of 40.32 (SD = 6.06) for mothers, and 43.43 (SD = 5.95) for fathers, and in the non-treatment group had a mean age of 41.09 (SD = 5.10) for mothers and 43.62 (SD = 6.75) for fathers. Table 1 contains information on educational level and income, as well as tests for differences between groups (non significant).

**Table 1:** Education and income by group.

	Treatment	Non-treatment	Chi Square (df)	p
Some higher education				
Mothers	14 of 49 28.6%	18 of 42 42.9%	2.024 (1)	.155
Fathers	23 of 46 50%	22 of 34 64.7%	1.718 (1)	.190
Monthly income				
>2000CHF	6	3		
2–4000 CHF	22	7		
4–6000 CHF	17	8		
6–8000 CHF	25	10		
8–10000 CHF	13	6		
>10000 CHF	17	8		
Total	100	42	.667 (5)	.985

### Measures

#### Child clinical diagnoses

The *Diagnostic Interview for Children and Youth for DSM-IV-TR*: *Parent Version* (Kinder-DIPS) [36] is a structured parent interview designed to assess mental disorders in children according to DSM-IV-TR criteria [34, 37] Clinician-based ratings of symptom frequency are assessed on a 4-point scale from 0 (never/seldom) to 3 (very often), with frequency ratings ≥ 2 judged as clinically relevant. Clinician-based ratings of the degree of distress and impairment (in home, school, friendship, and leisure domains) caused by the presenting symptoms for the child are provided on a 4-point scale from 0 (not at all) to 3 (very strong). Test-retest reliability (κ = 0.85–0.94; all DSM-IV diagnoses) and validity in past research are good, as are inter-rater reliability estimates for diagnoses of SepAD (κ = .85), overall diagnosis of an anxiety disorder (κ = 0.85), and other axis I disorders (κ = 0.85–0.94) [38, 39]. Interviews were conducted at baseline and post-therapy by trained clinical psychologists or advanced masters students, blinded to group status at all evaluations.

#### Parent anxiety

Parental pathology was assessed using self-report questionnaires and structured diagnostic interviews for both mothers and fathers. Parents completed the German version [40] of the Beck Anxiety Inventory (BAI) [40, 41] to assess manifestations of general anxiety. The BAI is a well-established 21-item self-report measure, with items assessing the severity of anxiety symptoms such as “nervous” and “lightheaded” during the past 7 days on a scale ranging from 0 (not at all) to 3 (strong). Scores are calculated by summing the responses for each item, for a final score ranging from 0 to 63. Cronbach’s alpha for the German version in the current sample was .90 for mothers and .87 for fathers.

#### Parent separation anxiety

Parent separation anxiety was assessed with 35 items from the Maternal Separation Anxiety Scale (MSAS) [42], which were translated into German [43], and also adapted for use with both parents. The full scale assesses parent separation anxiety, perceptions of negative effects of separation on children, and worry about separation while at work, using items such as “I feel worried when someone else looks after my child”, rated on a scale ranging from 1 (definitely not true) to 4 (definitely true). Three independent subscales focus on different aspects of maternal AS labeled (1) Maternal Separation Anxiety (MSA), (2) Perception of Separation Effects on the Child (PSEC), and (3) Employment Related Separation Concerns (ERSC). Sample items are “I don’t enjoy myself when I am away from my child” (MSA), “There are times in the lives of young children, when they need to be with people other than mothers” (PSEC), and “I would resent my job if it meant I had to be away from my child” (ERSC). Mean scores were calculated after reversing raw scores on all PSEC items and on four ERSC items (4, 15, 20, 30), with lower scores reflecting a lower level of maternal SA. Prior research indicates good construct validity, good internal consistency with reliabilities of .90, .71, and .79 for the subscales, and .88 for the total scale, and 3-month test-retest reliability of .73, .58, .72 (subscales), and .75 (full scale)[42]. The total scale score was used in the present study, with Cronbach’s alpha for the German version in the current sample of .88 for mothers and .89 for fathers.

#### Parent depression

Mothers and fathers also individually completed the German version [44] of the Beck Depression Inventory (BDI) [45] to assess manifestations of general depression. The BDI is a well-established 21-item self-report measure, with items assessing the severity of depression symptoms such as sadness during the past 7 days a scale which presents statements of increasing strength regarding the symptoms ranging from 0 (not at all) to 3 (strong). Scores are calculated by summing the responses for each item, for a final score ranging from 0 to 63. Cronbach’s alpha for the German version in the current sample was .85 for mothers and .82 for fathers.

#### Child separation anxiety

Parents completed the 12-item disorder-specific *Separation Anxiety Inventory for Children*: *Parent version* (SAI-P) [46] to assess the degree of their child’s avoidance of separations in a variety of settings (e.g., “I avoid going to sleep alone”) using a 5-point scale ranging from 0 (never) to 4 (always). The original 12-item SAI showed good reliability (internal consistency = .85; test-retest reliability .84) and construct validity [47]. In the present study, one item assessing experiences not typical for children in the younger age group (e.g., sleep-away camp) was dropped. Reliability for the 11 items in the current clinical sample were alpha = .73 (mothers), and .69 (fathers).

The *Parent Beliefs Questionnaire on Anxiety in Children* (PBQ-AC and PBQ-AC German Version)[48, 49] is a self-report measure assessing parents’ dysfunctional cognitions related to the anxiety of their child across several domains. Each of the 45 items is rated on a scale ranging from 0 (very untrue) to 10 (very true). High scores indicate high levels of dysfunctional beliefs. Authors of the scale hypothesized nine different factors: Outside world full of danger (e.g.: “The world is very unsafe for my child”); Child manipulates /is lazy (e.g. “My child manipulates me with his/her fears”); My child is weak (e.g. “My child is oversensitive”); Catastrophizing (e.g. “My child will grow lonely”); Conflict/anger (e.g. “Disagreement can damage the relationship between my child and me”); My partner/others (e.g.”My partner does not understand the needs of our child”); I must solve everything (e.g. “If my child cannot do something, it is better if I take over”); Powerlessness (e.g. “Nothing will help my child overcome his/her anxiety”); Over-identification/personification (e.g. “If my child is unhappy I have failed as a parent”). However, a factor analysis in prior research indicated a one-factor-solution [4]. The total score was calculated from an average of the 45 items. Internal consistency was high in the present sample (Cronbach’s alpha = .87 for mothers, .88 for fathers).

### Statistical analyses

To test sample differences in psychopathology levels at baseline, t-tests for independent samples were conducted comparing pathology levels between parents with children treated for SepAD and parents of healthy children receiving no treatment. Random coefficient models, a type of linear mixed model [50], with unstructured covariance structure, were used to analyze the parental outcomes variables (i.e., BAI, BDI, PSAS) assessed at baseline and 4-weeks post-treatment. Linear mixed models provide more efficient and less biased results than complete case analyses or analyses in which missing values are imputed using the last observation carried forward method [51]. Further, linear mixed models include participants with missing outcome values in the analyses, minimizing data loss, and increasing power. Our model included a random intercept as well as a random slope parameter when this improved model fit, based on Akaike’s Information Criterion, AIC [52]. A statistical model was set up for each measure for each parent (i.e., mothers and father separately) resulting in six models in all. The predictors were time (pre-, 4-weeks post-treatment), group (child treatment condition versus control condition), and time by group interaction. We calculated effect sizes for independent-groups pretest-posttest designs based on the descriptions by Feingold (2009) [53] for all outcome measures. Effect sizes were calculated using mean change scores for the treatment and the control group and standard deviations of the raw scores in baseline data [54]. We conducted analyses including on all available data from parents of all children initially randomized in the studies.

## Results

### Descriptive statistics and baseline differences

Means and standard deviations by group and time are presented in Table 2. Baseline t-tests indicated that baseline pathology levels were generally higher among mothers of children treated for SepAD than mothers of healthy children receiving no treatment. Significant group differences were found in mothers’ depressive symptoms (t = 3.19, p = .002, d = .64) and separation anxiety (t = 3.01, p = .003, d = .56). There was a trend level difference in mothers’ general anxiety (t = 1.82, p = .071). With regard to fathers, there were no significant differences in depressive symptoms (t = 1.49, p = .14) or general anxiety (t = 0.97, p = .33). There was a marginally significant difference in separation anxiety (t = 1.92, p = .06) between fathers with children treated for SepAD and fathers of healthy children receiving no treatment.

**Table 2:** Descriptive statistics by group and time.

	Baseline						4 weeks post					
	Treatment			No treatment			Treatment			No treatment		
	N	M	SD	N	M	SD	N	M	SD	N	M	SD
Mother												
Anxiety	101	7.75	7.64	41	5.27	6.7	76	4.43	5.56	23	3.04	4.78
Depression	101	6.64	5.45	41	3.71	3.49	70	4.63	4.56	22	3.55	4.23
Separation anxiety	100	2.23	0.30	42	2.06	0.31	76	2.08	0.31	22	2.02	0.32
Father												
Anxiety	87	4.91	5.47	30	3.80	5.12	62	3.00	3.44	15	1.67	2.19
Depression	87	4.44	4.09	30	3.17	3.83	64	2.58	2.89	15	1.87	2.33
Separation anxiety	88	2.18	0.31	32	2.06	0.34	62	2.01	0.33	14	1.96	0.29

### Mixed model effect of time (baseline vs. post-treatment)

There was a significant effect of time on both maternal and paternal levels of pathology, on all three tests: separation anxiety depression and general anxiety. Results indicated that symptoms of maternal and paternal pathology decreased from pre-treatment to post-treatment across groups.

### Mixed model effect of group (child treatment vs. control group)

Results of linear mixed models, presented in Table 3, with time (baseline, 4-week follow-up) and group (children treated for SepAD vs. healthy control group children without treatment) as fixed effects, indicated several significant effects. There were significant effects of group on maternal depression and separation anxiety, with a trend level effect on maternal anxiety. Further, there was a significant effect of group on paternal separation anxiety. Results indicated that mothers in the treatment group generally reported more overall pathology symptoms than mothers in the control group, and fathers in the treatment group reported more separation anxiety than fathers in the control group.

**Table 3:** Mixed model effects.

	Mothers			Fathers		
	Estimate B (SE), p	t(df)	95% confidence interval	Estimate B (SE), p	t(df)	95% confidence interval
Anxiety						
Intercept	7.7443(.7345), .000	10.54(140.26)	6.292–9.197	4.8692(.5760), .000	8.45(115.71)	3.7283–6.0102
Week	-.1408(.0353), .000	-3.99(129.50)	-.2106—.0710	-.0728(.0248), .003	-2.93(109.49)	-.1220- -.0236
Group	-2.4760(1.3673), .072	-1.81(140.15)	-5.1792-.2272	-1.0692(1.1401), .350	-.94(114.69)	-3.277–1.1892
Group*week	.0455(.0694), .514	.66(136.91)	-.0918-.1827	-.0097(.0519), .852	-.19(116.19)	-.1124-.0930
Depression						
Intercept	6.6110(.4704), .000	14.06(161.33)	5.6820–7.5398	4.4064(.4251), .000	10.85(130.11)	3.5645–5.2482
Week	-.0909(.0138), .000	-6.58(328.67)	-.1181—.0638	-.0628(.0136), .000	-4.63(75.75)	-.0898- -.0358
Group	-2.9036(.8773), .001	-3.31(160.70)	-4.6361- -1.1711	-1.2397(.8486), .147	-1.52(127.04)	-2.9207-.4413
Group*week	.0812(.0275), .003	2.95(338.05)	.0270-.1553	.0135(.0298), .639	.47(81.66)	-.0437-.0707
Separation Anxiety						
Intercept	2.2294(.0296), .000	75.40(154.06)	2.1710–2.2878	2.1877(.0328), .000	66.61(125.32)	2.1227–2.2570
Week	-.0070(.0010), .000	-7.01(195.86)	-.0089—.0050	-.0055(.0012), .000	-4.72(84.84)	-.0079- -.0032
Group	-.1660(.0548), .003	-3.03(151.69)	-.2743—.0577	-.1322(.0645), .043	-2.05(122.61)	-.2599—.0045
Group*week	.0055(.0020), .008	2.68(193.10)	.0015-.0095	-.0007(.0026), .788	-.27(72.86)	-.0060-.0045

Group coding: 0 = control, 1 = treatment. SE = Standard Error, df = degrees of freedom.

### Group by time interaction effect

**Fig 1:**
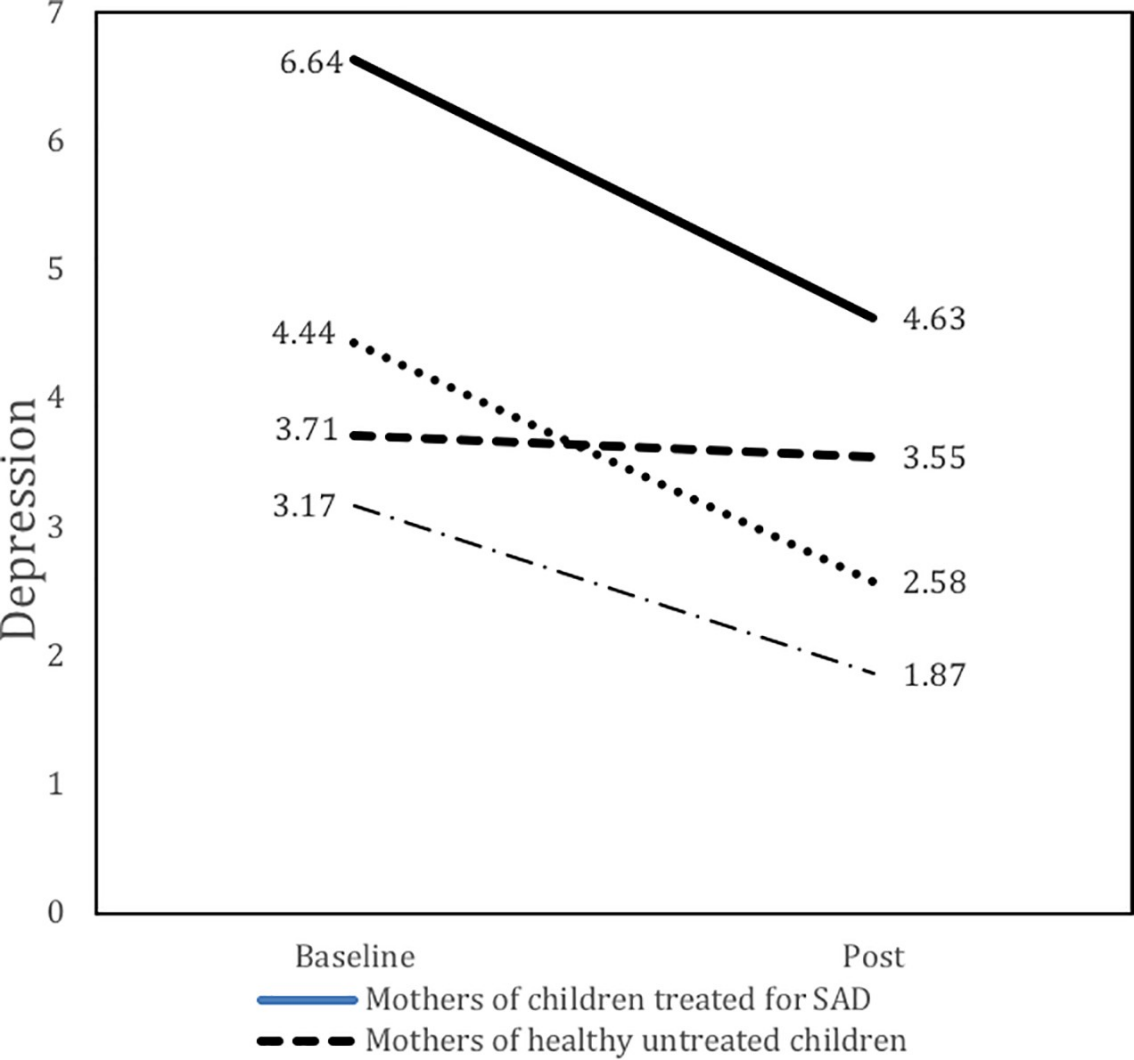
Parent depression by group and week.

**Fig 2:**
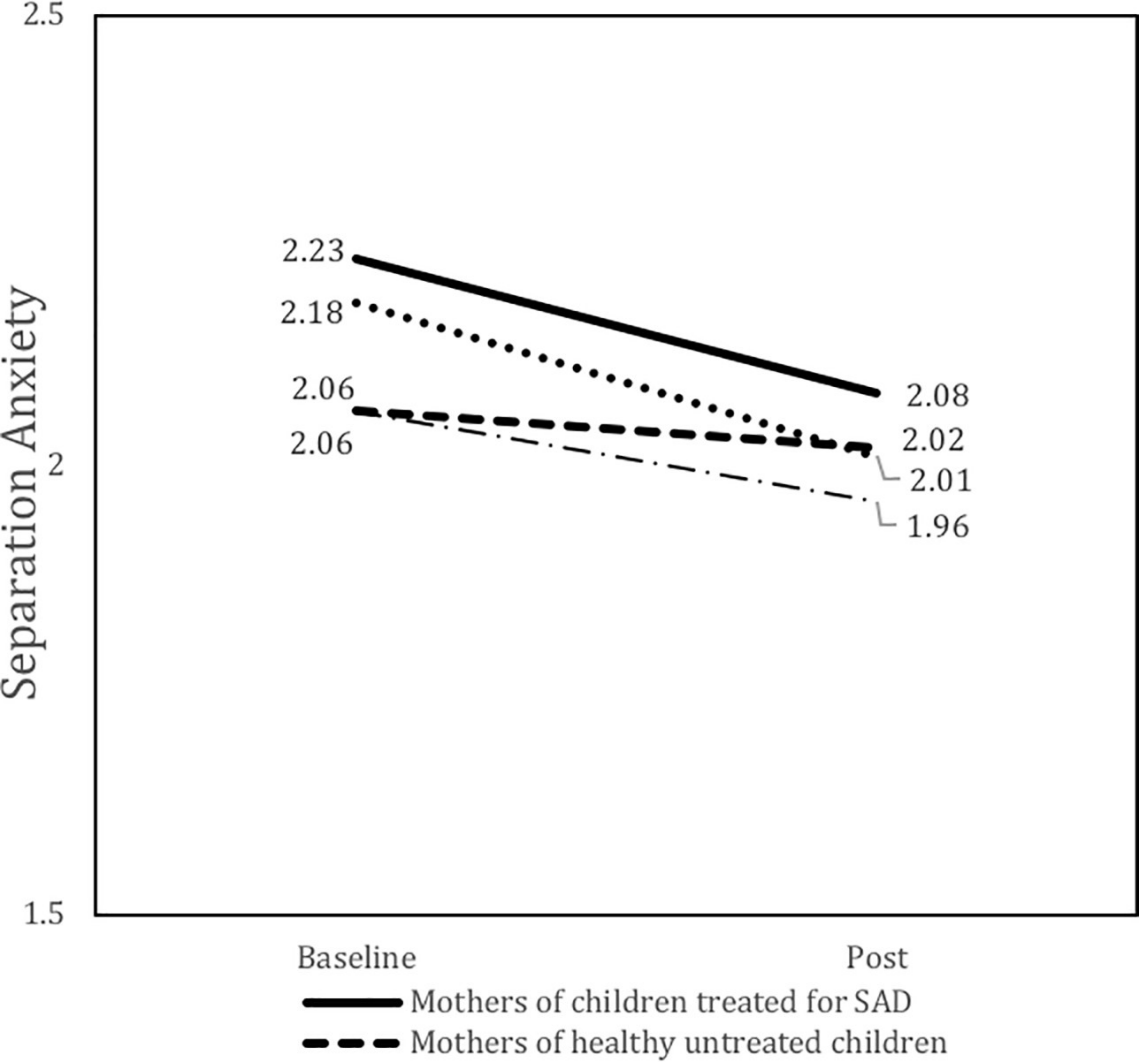
Parent separation anxiety by group and week.

There was a significant and medium size time by group effect (d = 0.32) on maternal levels of depression (Fig 1), indicating that the course of maternal symptoms of depression in the child treatment group differed from the course of symptoms in the control group. This interaction effect indicates that the decrease in maternal levels of depression was larger than what could be expected by time effects only. There was also a significant and medium size time by group effect (d = 0.39) on maternal separation anxiety (Fig 2), indicating that the course of separation anxiety symptoms among mothers of treated children differed from the course of symptoms in the control group. This interaction effect suggests that the decrease in maternal levels of separation anxiety cannot solely be explained by normal fluctuations due to time (e.g., spontaneous improvement). There was no significant time by group effect on maternal symptoms of general anxiety, indicating that the course of general anxiety levels of mothers of treated children was comparable to the course of general anxiety levels of mothers in the control group. Further, there were no significant time by group effects on any measure of paternal pathology, indicating that the course of symptoms among fathers of treated children was comparable to the course of symptoms among fathers in the control group. Post-hoc comparisons of levels of pathology at post-child-treatment, indicated that maternal levels of separation anxiety (t = -.68, df = 96, p > .05) and depressive symptoms (t = -.99, df = 90, p > .05) were comparable to levels of mothers of healthy children. As a last check on the models, all of the models were rerun controlling for type of therapy (TAFF or Coping Cat). This did not change the significance of the other effects, so they only the original models are presented here.

### Change score correlations

Post-hoc analyses examined the relationship between a change in parent-reported child separation anxiety and a change in that same parent’s self-reported anxiety, depression, and separation anxiety, and change in parents’ dysfunctional cognitions [4, 15]. Analyses also examined the relationship between change in parents’ dysfunctional beliefs and change in parents’ pathology. These analyses were conducted in the treatment group only, as the healthy group had too little baseline to post change to examine relationships between difference scores. Results (Table 4) indicated a significant positive correlation between change in father-reported child separation anxiety, and father’s self-reported own anxiety, such that a reduction in child separation anxiety was related to a reduction in father’s anxiety. Results also indicated a significant positive relationship between change in child separation anxiety and fathers’ dysfunctional cognitions, as well as significant positive relationships between change in mothers’ dysfunctional cognitions and mothers’ anxiety and separation anxiety.

**Table 4:** Correlation between parents’ report of child’s change in separation anxiety and parent’s own change in pathology (anxiety, depression, and separation anxiety) and dysfunctional cognitions, as well as correlations between change in parent’s pathology and dysfunctional cognitions.

	Change in parent’s own:						Change in child separation anxiety			
	Anxiety	Depression			Separation Anxiety		Mother report		Father report	
	R(p)	N	R(p)	N	R(p)	N	R(p)	N	R(p)	N
Change in child separation anxiety										
Mother report	-.129(.297)	63	.028(.836)	66	-.002(.998)	64				
Father report	.292(.041)	49	.033(.842)	49	-.082(.586)	47				
Change in parent’s dysfunctional cognitions (PBQ)										
Mother report	.292(.013)	71	.164(.192)	65	.320(.006)	72	.10(.434)	64	.258(.070)	50
Father report	.071(.616)	52	.221(.116)	52	.206(.128)	56	.098(.494)	51	.459(.001)	47

## Discussion

This study examined the transgenerational effects of two versions of cognitive-behavioral treatments for child Separation Anxiety Disorder (SepAD) on the mental health of parents. A large body of research has demonstrated the efficacy of CBT programs on child anxiety in general [9] and child SepAD in particular [16, 17]. Elevated levels of parental anxiety and depression tend to co-occur with anxiety disorders among children [24]. However, relatively little is known regarding the potential beneficial effects of child treatments on mental health of parents of children with anxiety disorders. The present study investigates pre- and post-treatment differences before and after participation in evidence-based CBT for children on symptoms of separation anxiety, general anxiety, and depression among mothers and fathers of children treated for SepAD. Strengths of the present study include the relatively large sample size as combined from two clinical trials, and the use of data from clinical trials where the child treatment efficacy was established.

Results showed significant improvements in symptoms of separation anxiety and depression among mothers of children treated for SepAD, post-child-treatment, in comparison to mothers of healthy children. Compared to the pre-treatment assessment, symptoms of separation anxiety and depression were significantly lower at post-treatment assessment for mothers of children with SepAD. Remarkably, post-hoc comparisons indicated that maternal levels of separation anxiety and depressive symptoms were comparable to levels of mothers of healthy children after child treatment. No significant effect of child treatment across time was found in the level of general anxiety among mothers. A possible explanation for these differential effects may be floor effects in that that child treatment can only reduce pathology, when pathology levels are not already near the floor at baseline. However, in the present study, mothers of children with SepAD and mothers of healthy children did not significantly differ in general anxiety at baseline, while baseline differences existed in separation anxiety and depressive levels, which were significantly reduced in the treatment condition compared to the control condition. For mothers, change in child separation anxiety was not related to change in parent pathology in mothers in the treatment group. That is, despite some baseline-to-post effects, these changes were not directly related to change in child anxiety. They may have been due, rather than to actual child change, to nonspecific treatment factors such as hope or the emotional/social support provided by having the child in treatment, to transfer of CBT skills themselves to the mother For fathers, no significant baseline-to-post effects were found among fathers of children treated for SepAD on any measure of paternal pathology. Possibly, this may be attributable to low statistical power, as the sample size of fathers participating in the present study was lower than the sample size of mothers participating in the present study. When interpreting the effect sizes rather than the significance values, one small effect (d = .28) emerged for paternal separation anxiety. That is, fathers of children treated for SepAD showed a larger reduction (d = .55) in separation anxiety than fathers of children without SepAD (d = .27). Importantly, father-perceived change in child separation anxiety was significantly associated with change in the same direction in father’s own self-reported anxiety. Similarly, father-reported change in child separation anxiety was significantly associated with father’s dysfunctional cognitions. Generally, these findings were in line with the initial hypotheses and with the result observed in prior research, indicating a post-child-treatment reduction in mothers’, but not necessarily fathers’, psychopathology [33], but also pointed to the potential for actual child change in separation anxiety to be associated with father’s own anxiety and dysfunctional cognitions. <Although fathers do not show general improvements in mental health after child treatment,> they do show improvements in general anxiety levels and dysfunctional cognitions following perceived improvement in their child’s anxiety. A possible explanation for this difference between mothers and fathers is that fathers are typically less involved in the treatment of their children, and thus would not benefit as much from spill-over effects or non-specific treatment factors (i.e., support or hope). For fathers, actual changes in their child's behaviors may be necessary for anxiety and cognitions to improve. Finally, post-hoc analyses did indicate that change in mother’s dysfunctional cognitions was related to change in mothers’ anxiety and separation anxiety, indicating that improvements among mothers occured both on an behavioral and cognitive level

Based on social learning theories, we expected to find a transgenerational relationship between child psychotherapy and parents’ pathology. Previous studies have already reported effects of interventions for parents on mental health and health behavior of children [29] and initial evidence also suggests transgenerational treatment effects from children to parents [31, 32]. Up to this point, studies examining child treatment effects on parents have focused on children with ASD, while transgenerational effects of treatment for children with a primary diagnosis of an anxiety disorder is a topic rarely explored [33]. Previous work on therapy for child separation anxiety indicates secondary effects of child SepAD treatment on dysfunctional cognitions of parents [17]. Here, a reduction in dysfunctional beliefs of parents was found after child treatment. However, the study only compared two treatment conditions and did not include a control group to control for naturally occurring time effects. Although findings indicate a decline in dysfunctional cognitions among parents, the effects of child treatment on parental mental health have not yet been examined. The present study fills this gap by illuminating a potential relationship between child treatment and maternal pathology, and child improvement and father improvement. Interestingly, baseline-post reduction in pathology levels were found only when pathology levels were elevated before child treatment, indicating that some level of pathology is prerequisite for child treatments effects to be visible and not subject to a floor effect. However, the mechanisms by which child treatment effects operate among parents are not well understood. It is yet unclear whether improvements in the mental health of mothers are due to improvements in separation anxiety in their children. Alternatively, it is possible that non-specific treatment effects (i.e., placebo effects in psychotherapy such hope or confidence) may explain the reduction in maternal pathology levels. However, it is unlikely that specific treatment effects produced changes in the mental health of mothers, as improvements were observed in mothers of children in the child-focused treatment. The present findings are correlational in nature and do not include a true control group (i.e., a group of parents of children diagnosed with SepAD, who did not receive any treatment). However, they provide some insight in that they show that mothers whose children were in therapy improved in their own symptoms of psychopathology. Whether these improvements were due to unspecific therapy effects or child effects is unclear and more systematic research is needed.

The present study has several limitations. First, groups in the present study differed in overall mean levels of symptoms at baseline (i.e., the parental control group had lower psychopathology scores than parents of children receiving treatment), and it is possible that the comparisons (parents of healthy children) in the present study did not decline much over the course of the study, because their symptom levels were already lower to begin with (i.e., “floor effect”). However, the quasi-control group gives first evidence that the change in depression and separation anxiety in mothers of SepAD may not be solely a time effect. Second, children who received TAFF and Coping Cat therapy were combined in the present study to increase statistical power. Although prior research indicated no difference in child outcomes between these two therapies, the TAFF-program includes not only child treatment, but also parent and family sessions. Although treatment of parental psychotherapy is not the direct aim of these sessions, the inclusion of parents in the child session could have produced direct effects on parental mental health. However, when type of therapy was included in the models, it didn’t have an effect and didn’t change the significance of the other effects. Third, the present study lacked a clinical control group. The study was not specifically designed around the question of transgenerational effects, and data on parent pathology are only available at baseline and post-treatment. Thus, parent pathology data were not collected at post-waitlist. If they had been, this would have allowed for a clinical control comparison (i.e., comparing parents of children with SepAD in immediate versus delayed treatment conditions). The lack of a clinical control group receiving delayed or no treatment or alternative treatment (i.e., medication) or isolated therapy components (i.e., cognitive therapy only, exposure only) means that it is not possible to identify the mechanism of the effect in this study. The effect could be due to transfer of knowledge between children and parents, parents’ direct participation in therapy, reduced strain on parents as a result of having a healthier child, or simply due to time combined with a greater room for improvement in the treatment mothers. A clinical control group may help to answer these questions in the future. Fourth, parental psychopathology was assessed and evaluated dimensionally in the present study, and results do not provide information on whether parent clinical disorder status may change in conjunction with child treatment. Finally, it is not possible to determine the order of effects (cause-effect) in the present study. We measure change in parents and children at one time point (post-measurement), so we cannot infer whether change occurred first in children or parents. It is possible that the effects are interactive. Other designs with multiple measurements during the course of treatment would be necessary to determine the order of effects.

The present study has clinical implications. First, it underlines the importance of the association between challenging child behavior and the mental health parents, including symptoms of depression, anxiety, and stress. There is a need to address these challenges for parents. In addition, this study shows that child treatment for separation anxiety is associated with a reduction in symptoms of depression and separation anxiety among mothers, indicating that child treatment (with or without family sessions), and that change in child separation anxiety is related to change in fathers’ own anxiety. This study may be considered a first step in understanding a stepped-care approach towards mental health, which may decrease psychological distress and prevent manifestation of clinical disorders among parents.

The present findings provide a first look into the association between child therapy for anxiety and mental health of parents, showing that child psychotherapy is associated with more rapid decreases in mothers’ own separation anxiety and depressive symptoms, when compared with mothers of healthy children not undergoing psychotherapy. The exact mechanism underlying this effect is not known, and further study is necessary to disentangle them. A future study in this area may compare parents of children undergoing psychotherapy to parents of clinical control children on a waiting list to receive psychotherapy, and parents of clinical control children receiving alternative treatment and/or isolated components of treatment, and examine correlations between degree of child recovery and degree of parent recovery, to determine whether parents’ recovery is spontaneous, due to therapy knowledge transfer, or due to the child’s recovery. Future research should also include a population study to examine the relationship between change in child and parent pathology in both families with disordered and non-disordered children, on a larger scale.

In conclusion, the present exploratory study showed that child SepAD is associated with symptom of depression and anxiety among mothers and that treatment for child SepAD may have positive effects on reducing maternal levels of separation anxiety and depression, though the mechanisms are yet unknown. It also showed a small to medium correlation between change in father-reported child separation anxiety and father’s own anxiety in families with children diagnosed with SepAD and undergoing treatment. This study indicates that child psychotherapy may be related to improvement of mental health in mothers, and child improvement itself may be related to father’s own improvement, which may be particularly important when parents’ own pathology levels are high.

## Supporting information

S1 File Manuscript data. (SAV)

S2 File Manuscript data transposed. (SAV)
